# Nonpharmacologic Interventions for the Self-Management of Anxiety in Parkinson's Disease: A Comprehensive Review

**DOI:** 10.1155/2019/8459579

**Published:** 2019-05-02

**Authors:** Susan K. Chandler, Jo Lynne Robins, Patricia A. Kinser

**Affiliations:** Virginia Commonwealth University School of Nursing, Virginia, USA

## Abstract

Anxiety in Parkinson's disease (aPD) is underdiagnosed, undertreated, and understudied. As many as 50% of persons diagnosed with Parkinson's disease (PD) are reported to suffer from anxiety. Current treatment is largely pharmacologic, which can result in a myriad of undesirable and unsafe side effects. The aim of this paper is to examine intervention studies of self-managed nonpharmacological strategies for the treatment of anxiety. A comprehensive review was conducted on experimental or quasi-experimental trials that included self-management approaches for the nonpharmacologic treatment of anxiety as a primary or secondary aim or outcome measure. Thirteen studies were identified from four databases. Study quality demonstrated variability in design and delivery of self-managed interventions; sample sizes were small; anxiety was most commonly a secondary aim; and the use of anxiety measures varied widely. Statistical significance was evident in slightly more than 50% of the anxiety intervention studies. A common element in the interventions in all studies was the focused use of breath. Further research is needed to determine the feasibility of using focused breathing, alone, as an intervention for the self-management of anxiety in Parkinson's disease.

## 1. Introduction

Parkinson's disease (PD) is the second most common neurodegenerative disorder, surpassed only by Alzheimer's [1]. In the United States, an estimated 1 million people suffer from PD, with about 60,000 new cases diagnosed annually. PD affects all racial and ethnic groups in both genders, with men at a slightly higher risk [2]. The economic impact for direct and indirect medical care costs exceeds $20 billion annually in the United States alone, including time lost from work for patients and caregivers and disability payments to patients [3, 4].

PD is a chronic, progressive disease associated with a severe loss of dopaminergic neurons in the substantia nigra and is characterized by motor symptoms such as bradykinesia, resting tremor, and rigidity, with progressively worse postural stability [5, 6]. While these motor symptoms (MS) are assumed to be the most disruptive, medications and surgery have been successful management options [7]. PD is also characterized by non-motor symptoms (NMS) such as bladder issues, constipation, drooling, fatigue, loss of smell, orthostatic hypotension, rapid eye movement (REM) sleep disorder, and cognitive and psychiatric disturbances, including anxiety and depression [3, 8]. These symptoms have a stronger association with reduced quality of life (QOL) than MS [9] and can lead to placement in a long-term care facility [4]. As with MS, there have been a variety of medications, treatments, and interventions identified to manage many of the NMS, yet many symptoms remain difficult to treat.

Anxiety is considered to be one of the most distressing and difficult-to-treat NMS in PD [10, 11]. Anxiety affects as many as 50% of persons with PD and can occur at any stage of the disease [12, 13]. Potential etiologies of anxiety in PD include Parkinson's-related neurodegeneration, reaction to the diagnosis of PD, or as sequelae of dopamine replacement therapy [4, 5]. Anxiety is particularly worthy of study because unlike other NMS, it directly affects individuals with PD by worsening MS such as tremors, gait problems, dyskinesias, freezing, and postural instability [10]. Episodes of anxiety result in avoidance from social situations, thus negatively impacting QOL [14, 15]. Anxiety also interferes with onset and return to sleep during the night resulting in excessive daytime sleepiness [16, 17].

Pharmacologic treatment of anxiety is common yet problematic for individuals with PD. Antidepressants and anxiolytics, such as selective serotonin reuptake inhibitors (SSRIs), selective norepinephrine reuptake inhibitors (SNRIs), tricyclics, antipsychotics, and benzodiazepines, comprise the mainstay for treating anxiety [18–21]. However, use of these medications in individuals with PD may result in an upsurge in daytime somnolence, cognitive dysfunction, confusion, hallucinations, and falling related to existing postural instability and gait issues [19, 22]. Further, the evidence is inconclusive regarding efficacy of antidepressants for treating anxiety [11]. Coakeley et al. [18] claimed that SSRIs were designed for the treatment of depression and MS, not anxiety, and pharmaceutical trials have not adequately measured their efficacy for the treatment of anxiety. Additionally, studies in the literature indicate that anxiety does not respond as well to psychotropic medications, such as quetiapine, when compared to the general population [20, 21].

Clearly, safe, efficacious nonpharmacologic approaches are needed for individuals with PD who experience anxiety. One such approach may be the use of self-management strategies. Self-management has been defined in numerous ways in the literature [23–26]; for the purpose of this review, self-management is defined as the ability of the individual (with or without help of the family and community), combined with the oversight of healthcare professionals, to manage a combination of symptoms, treatments, and recommendations for lifestyle modifications and emotional management of one or more chronic diseases [23]. Self-management employs a complex framework that includes facilitators, barriers, management processes, and proximal and distal outcomes [27]. In 2011, the National Institute of Nursing Research (NINR) recommended as a part of their strategic plan that the science of self-management be supported as a priority, with the goals of developing self-management approaches to reduce the burden of chronic illnesses and improving quality of life [28].

A growing body of literature suggests that self-management strategies can be used successfully in PD. Guidelines for the development of a PD self-management training program using a theoretical orientation were described in a review by Lyons [29], who suggested that the goal of self-management interventions for chronic diseases is often enhancement of QOL and function. In PD, the patient is ultimately responsible for daily management of their PD symptoms, and healthcare professionals serve as an integrative team to provide resources, support self-efficacy of the patient, and evaluate outcomes [29]. Sajatovic et al. [30] conducted a randomized controlled trial (RCT) of thirty patients examining the self-management concepts of education and health coaching to address depression in PD and found that depressive symptoms improved significantly. Yorkston et al. [31] qualitatively explored patient perspectives of PD-associated dysarthria and concluded that incorporating self-management principles into a traditional dysarthria intervention would result in additional tools for them to work with as well as bolstering their self-efficacy.

Although there have been studies incorporating self-management for several NMS in PD, it is unclear if self-management of anxiety is effective. Thus, the purpose of this review is to identify studies using self-management or components of self-managed nonpharmacological interventions for the treatment of anxiety in individuals with PD.

## 2. Methods

A comprehensive literature search was performed to identify existing evidence of self-managed nonpharmacological studies of anxiety interventions for individuals with PD. Using the search terms “Parkinson disease,” “Parkinson's disease,” “Paralysis agitans,” “Anxiety,” “Depression,” “Interventions,” “Treatments,” and “Self-management,” a search of the literature was performed using the Cochrane Database of Systematic Reviews, CINAHL, PubMed, and PsycARTICLES databases. To ensure rigor, only experimental and quasi-experimental studies were included. Additional limits were English language and publication dates between June 1997 and June 2017. Exclusions were deep brain stimulation (DBS) or similar neurosurgeries, pharmacologic management of anxiety and dementia, and individual case studies. Articles investigating anxiety as primary aims, secondary aims, or outcomes were included. Appraisal was based on the Johns Hopkins Evidenced-Based Practice Research Evidence Appraisal model [32].

## 3. Results

**Figure 1:**
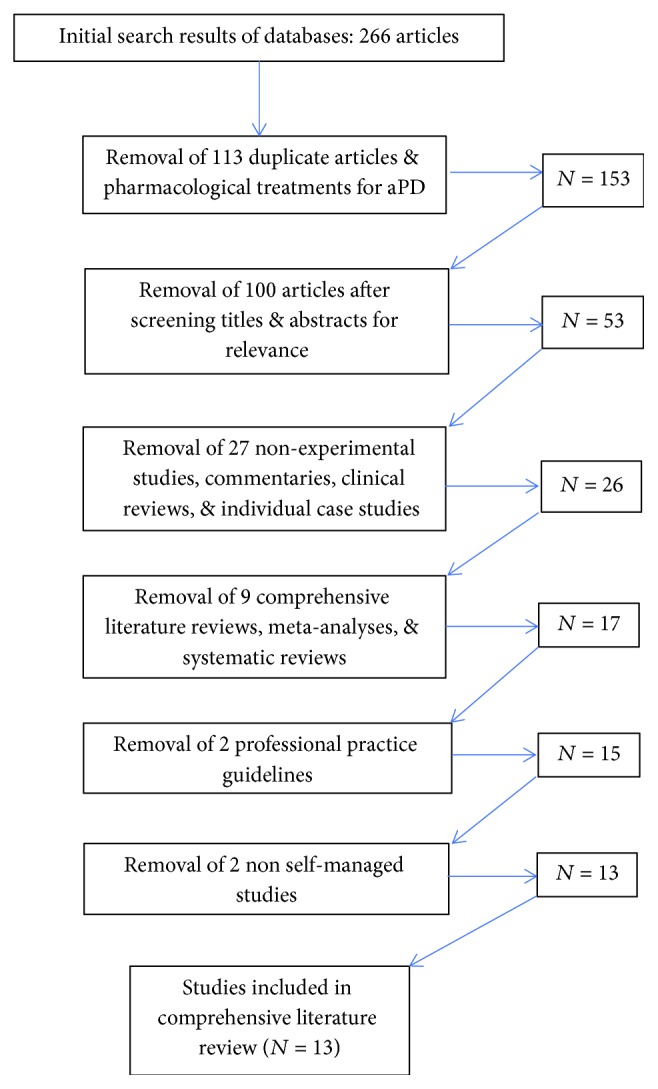
Consort table of comprehensive literature search.

The initial search resulted in a total of 266 articles. Removal of duplicates and pharmacological treatments yielded 153 studies. The titles and abstracts of the remaining articles were screened for relevance, with an additional 100 removed. Twenty seven articles that were nonexperimental studies, commentaries, clinical reviews, or individual case studies were eliminated. Nine comprehensive literature reviews, including meta-analyses and systematic reviews, were removed after screening references for additional potential studies. Two practice guidelines for the treatment of anxiety in PD issued by neurology professional organizations were also removed, as their recommendations for the treatment were inconclusive due to the paucity of evidence. Two studies were excluded because they evaluated interventions that could not be considered self-managed (e.g., acupuncture); thus, they were excluded. The final analysis revealed thirteen nonpharmacological studies containing components of self-management in their interventions (Figure 1). Authors, study design, purpose, intervention, sample size, and statistical significance are summarized in Table 1.

### 3.1. Study Characteristics

Overall, there were five experimental and eight quasi-experimental trials. Three quasi-experimental studies focused on anxiety as the primary aim. The remaining ten studies measured anxiety as a secondary aim or as a proximal outcome. In addition to anxiety, other research outcomes were depression (six studies), function and well-being, quality of life, and MS and NMS. The most commonly evaluated intervention was cognitive-behavioral therapy, administered in a variety of delivery settings (individual, group, or via the telephone) (in *n* = 9 studies). Sample sizes ranged from 7 to 80 participants, with the mean number of participants across all studies as twenty-five. Numbers reported for the studies reflect those participants completing the interventions and included in the statistical analyses. PD stage was not described for the participants in any study. Two experimental and four quasi-experimental trials demonstrated statistically significant results. A fifth quasi-experimental trial measured anxiety with 3 different tools; only one indicated significance. The studies are organized as to experimental or quasi-experimental, with statistically significant results reviewed first in each group.

### 3.2. Experimental Studies

Among the five RCTs, two resulted in statistically significant findings for improving aPD. Modified CBT plus clinical monitoring (compared to clinical monitoring alone) was used in the first RCT (*N* = 80) with significant results [33]. Authors looked at anxiety as the secondary aim (depression was the primary aim). The ten-week CBT intervention included exercise, behavioral activation, thought monitoring, relaxation training, sleep hygiene, and worry control. The Hamilton Anxiety Rating Scale (HAM-A) [34] was used to measure changes in anxiety. Results indicated significant reductions in anxiety (*p* < 0.0001) and depression (*p* < 0.01) immediately post intervention. This effect was sustained at 6 and 12 weeks.

The second RCT (*N* = 32) with significant results compared the effectiveness of listening to relaxing music (control group) to a massage intervention known as neuromuscular therapy (NMT) over 4 weeks [35]. Anxiety was measured as a secondary outcome. The Beck Anxiety Inventory (BAI) [36], a widely used anxiety inventory, was used to measure anxiety. While both interventions reduced anxiety when measured immediately post intervention (music, *p* = 0.002; NMT, *p* = 0.0009), only the group listening to relaxing music sustained a significant improvement when measured again eight days post intervention (*p* = 0.002).

The remaining three RCTs did not result in statistically significant outcomes. In the first of these, a 12-week exercise program designed specifically for PD was compared to a chronic disease self-managed program including exercise (*N* = 24) [30]. Changes in anxiety (secondary aim) were measured using the Covi Anxiety Scale (COVI) [37], a 3-item tool based on the patient's verbal report, behavior, and somatic symptoms. There were no significant changes in anxiety immediately following the intervention (12 weeks, *p* = 0.089) or at long-term follow-up (24 weeks, *p* = 0.587).

In the second nonsignificant experimental trial, effects of a six-week mindfulness-based intervention using a dyad of patients and caregivers was compared to a waitlist control group (*N* = 57) [38]. The Depression Anxiety Stress Scale-21 (DASS-21) [39] was used to measure anxiety as a secondary outcome. No statistically significant results were demonstrated post intervention (*p* = 0.54) or at the six-month follow-up (*p* = 0.33).

The final RCT compared the effects of tele-based CBT on anxiety and depression in PD to usual care plus support (*N* = 7) [40]. The primary aim of the 8-week program was to pilot the feasibility of identifying patients with anxiety and depression, with a secondary aim of determining the effectiveness of telephone-based CBT for their NMS. As measured by the BAI, there was no significant change in anxiety; however, a medium effect size was noted post treatment (eta^2^ = 0.08) followed by a large effect size (eta^2^ = 0.5) one month post treatment indicating a notable difference between the two groups. The authors concluded that CBT delivered via the telephone may be useful in identifying psychiatric NMS.

### 3.3. Quasi-Experimental Studies

Eight uncontrolled studies using self-managed nonpharmacological interventions for anxiety were identified in the literature. As with the experimental RCTs, they demonstrated mixed results. The first quasi-experimental study used a form of telephone-based CBT as an intervention for treating anxiety as a secondary aim (*N* = 20) and required participants to attend twice weekly classes for 5 weeks [41]. At the conclusion of the classes, improvements in anxiety and negative thoughts were significant (*p* < 0.01), as measured by the HAM-A, and remained significant when measured again at the one-month follow-up.

The efficacy of an 8-week group CBT program on anxiety and depression as compared to a waitlist control group was examined in the second quasi-experimental trial (*N* = 18) [42]. Originally designed as a RCT, problems with recruitment resulted in changing the study design to a nonrandomized trial. The CBT program included psychotherapy, psychoeducation, relaxation training, cognitive therapy, and problem-solving. Also included was content specific for PD, such as activity scheduling, anxiety triggers, and the fear of falling. As measured with the DASS-21, significant reductions in anxiety and depression (*p* < 0.001 and *p* = 0.001, respectively) were evidenced in the treatment group. These effects persisted at both 1 and 6 months post intervention.

The third quasi-experimental study examined MS and NMS, including anxiety, using 90-minute group CBT classes taught over 12 weeks (*N* = 7) [43]. Content specific to PD such as avoidance of social situations, fear of the future, and the understanding of emotions experienced in PD were included in the standard CBT program. At the conclusion, the change in anxiety, measured by the HAM-A, was significant (*p* = 0.027).

In the fourth quasi-experimental study, a 6-week group mindfulness intervention was employed (*N* = 14) [44]. The primary aim was reduction in anxiety. Based on results from the Geriatric Anxiety Inventory (GAI) [45], which is a self-report measure of anxiety, immediate postintervention scores were significant (*p* = 0.03); however, results were not sustained when measured at 6 months out (*p* = 0.20).

The aforementioned quasi-experimental study was followed a year later with a manualized and tailored 6-week CBT intervention with booster classes at 3 and 6 months from baseline [46]. The primary aim was to measure postintervention and sustained effects of the intervention on anxiety by using a dyad of patients and caregivers (patients *N* = 12; caregivers *N* = 10). Investigators hypothesized that the inclusion of caregivers would reduce caregiver burden while improving their well-being, due to the high dependence of PD patients on others. Investigators used three anxiety tools: the GAI [45], a self-report tool; the HAM-A [34], an observer-rated measure of clinical change in response to treatment; and the Informant Questionnaire for Anxiety in Dementia (IQAD) [47] which is completed by caregivers. Patients experienced a reduction in anxiety when measured by the HAM-A at program completion, which was significant at all outcome time points (post intervention *p* = <0.01; 3 months *p* = 0.02; 6 months *p* = 0.04). The GAI was not significant post intervention (*p* = 0.08); however, it was statistically significant at 3 and 6 months (*p* = 0.02; *p* = 0.05), post intervention. The IQAD did not indicate significance post intervention (*p* = 0.44) or at the 3- and 6-month follow-up (no data).

The remaining three quasi-experimental studies using self-management nonpharmacologic interventions did not yield statistically significant differences in anxiety. A modified program of CBT was conducted over a course of 10-14 sessions providing individualized treatments in person (*N* = 15) [48]. The number of sessions (mean number of sessions was not reported) was based on need, determined at the time of enrollment. Relaxation training was a major emphasis of this study. Change in anxiety was measured incidentally (not as an aim) using the Spielberger State-Trait Anxiety Inventory (STAI), the most widely respected and used measure of general anxiety [49]. Results of the study were nonsignificant (*p* = 0.065).

The second nonsignificant quasi-experimental trial used individualized CBT delivered either via telephone or in person (compared to enhanced usual care) in an 8-session intervention (anxiety secondary aim) delivered over 12 weeks (*N* = 16) [50]. The majority of the participants choose to participate in sessions by telephone, with resources for anxiety, depression, and healthy living (specific to PD) included in all CBT sessions. The Structured Interview Guide for the Hamilton Anxiety Scale (SIGH-A) [51], a guide developed to standardize the questions used in the original HAM-A tool [52], was used to measure anxiety. Though not statistically significant (*p* = 0.11), between-group effect sizes for change scores were 1.44 for anxiety when measured at both baseline to post treatment and baseline to 1-month follow-up. Despite the absence of significant improvements in study outcomes, the intervention was deemed to be feasible for individuals with anxiety.

The final quasi-experimental study deemed nonsignificant was of Japanese persons with PD and used “manga,” a widely accepted educational approach employing comic novels [53]. Nineteen participants attended 6 weekly classes. Investigators examined anxiety as a secondary outcome in this study and used culturally adapted CBT. Three measures of anxiety were used: the Hospital Anxiety and Depression Scale (HADS) [54], STAI [49], and Overall Anxiety Severity and Impairment Scale (OASIS) [55]. Results showed medium effect sizes when measured by each of three anxiety tools. However, the linear mixed model did not indicate statistical significance (*p* = 0.27, *p* = 0.48, and *p* = 0.79).

## 4. Discussion

The aim of this literature review was to identify existing studies employing nonpharmacologic self-management strategies for the treatment of anxiety in PD. A total of thirteen studies were included in this review; however, only 4 identified treatment of anxiety as the primary aim. Anxiety is recognized as a predominant NMS of PD that results in a decline in quality of life [4, 18]. Dissanayaka et al. [12] and Erro et al. [13] reported that as many as 50% of those diagnosed with PD endorse experiencing anxiety as a NMS. Considering the prevalence of anxiety, the acknowledged safety issues with pharmacological treatments, and the overall negative impact on QOL resulting from anxiety, the small number of studies with anxiety as a primary aim indicates a serious gap in research.

Results of the identified experimental and quasi-experimental studies were mixed. Of the five experimental studies, two were statistically significant. Five of eight quasi-experimental studies displayed significant results, while the remaining three did not reach statistical significance. Conclusions about the efficacy of the interventions as alternatives to pharmacological approaches for the management of anxiety are difficult to draw, given multiple *methodological issues*. Of the thirteen studies included in this review, the majority of *sample sizes* (11 of 16) consisted of 21 participants or less. The largest sample size included 80 participants.

Another issue related to rigor was the *variability in the instruments* used to measure anxiety. The HAM-A, including use of the structured interview guide for the HAM-A (SIGH-A), was used in 5 studies. However, overall, ten different measures of anxiety were used in the 13 studies included in this review. The tools measured a variety of different anxiety states, such as social anxiety, panic disorder, episodic anxiety events, and generalized anxiety disorder, with some of the measures relying on observations of caregivers and somatic complaints which frequently are confused with PD symptoms [56, 57]. In addition to variability, there was an overall lack of documented reliability of the instruments used to measure anxiety. Seven of the thirteen studies made no mention of the psychometric properties of the anxiety measures used. Troeung et al. [42] included a general statement attesting to the validity and reliability of the anxiety measures they utilized without specifics, as did Dissanayaka et al. in both of their studies. While Calleo et al. [50] did include Cronbach's alpha (0.78) for the SIGH-A tool, the most comprehensive information on psychometric properties of the anxiety tools were detailed in only two of the studies, Veazey et al. [40] and Shinmei et al. [53].

There was also significant *variability in the design and delivery of SM nonpharmacological interventions*. There were a total of 5 different interventions across the 13 studies. The most consistently used was CBT (5/13); however, there was little consistency in its design and delivery (Table 1). Further, *intervention fidelity* was not documented in any of the studies. These methodological issues make it difficult to validate, synthesize, and translate findings from this literature review.

To further emphasize this point, two neurological professional organizations conducted reviews intended to update practice guidelines for the management of anxiety. The Movement Disorder Society (MDS) Task Force on Evidence-Based Medicine (EBM) review was updated in 2011, extending their original review to include NMS. Unfortunately, they were not able to identify any RCTs for the management of anxiety that met their inclusion criteria. Citing insufficient evidence, they were unable to make any recommendations [11]. Likewise, the Quality Standards Subcommittee (QSS) of the American Academy of Neurology (AAN) evaluated treatment options for NMS of PD [22] and also concluded that data were insufficient to make recommendations for the treatment of anxiety.

The addition of self-management approaches may be a potential strategy for the development of interventions to improve control of anxiety. An Institute of Medicine [58] report stated that effective self-management programs not only imparted responsibility to the patient but also provided them tools for use in their care. Grady and Gough [59] suggest that models of self-management be modified to the specific chronic disease. There is mounting evidence that tailoring self-management interventions for persons with diabetes, obesity, chronic pain, and arthritis have resulted in successful clinical outcomes, for example [60–63].

The challenge becomes identifying what intervention or interventions would be appropriate for the study of anxiety experienced by those with Parkinson's disease. Nine of the thirteen studies included in this review used CBT, with each study using a slightly different combination of therapeutic approaches. To this end, most of the CBT approaches shared the use of focused breathing to slow and redirect thoughts in conjunction with the physiological response of relaxation.

Breath has been used as a primary intervention in a number of clinical studies, including anxiety, depression, and stress, and for relief of somatic symptoms of chronic diseases [64–69]. No studies were identified using focused breathing as a stand-alone intervention for the treatment of anxiety in Parkinson's disease, however. As an intervention, focused breathing is accessible and safe, requires only a minimal investment of time and practice to become competent when taught by an experienced practitioner, and clearly falls within the category of self-management. Determining the acceptability and feasibility of focused breathing as an intervention for the self-management of anxiety in Parkinson's disease by conducting a pilot study would appear to be a worthwhile endeavor. Currently, the Institutional Review Board (IRB) of the authors' academic medical center has approved such a study and recruitment will begin shortly.

This comprehensive review evidences that additional well-designed research is needed and also informs directions for future research that build on the existing evidence base. First, each experimental and quasi-experimental study used at least one component of self-management among their interventions. Compared to patient education, which conveys knowledge of the disease and its process, self-management is a health-promoting behavior, which adds skills, resources, and the self-efficacy needed to stimulate change [29]. Consistent use of a self-management theoretical framework (e.g., The Individual and Family Self Management Framework [27]) could guide study design, intervention design, and delivery as well as study outcomes. Second, attention to methodological rigor is of paramount importance including experimental design, adequate sample sizes, consistent use of reliable and valid instruments, and monitoring and reporting of intervention fidelity. Third, while sample sizes were small, there was high retention of participants across studies providing some evidence that self-managed nonpharmacologic approaches are feasible and acceptable. That said, attention to potential barriers could increase sample size and further foster retention. For example, it should be noted that most interventions in this review required attendance at numerous sessions, often more than once a week, making transportation a potential barrier to participation because persons with PD often must rely on others for transportation as their disability increases as well as due to transitioning to an “off” state, which renders mobility extremely difficult. Further, in several of the studies, enrollment required the availability and participation of either a family member or friend. The presence of these barriers and time off from work are significant considerations in the design of future research for this population.

## 5. Conclusion

Anxiety is a major contributor to decreased quality of life for those living with PD. Existing research for effective nonpharmacological interventions for the management of anxiety is sparse and lacks rigor. Clearly, more attention should be given to finding a solution for this troubling NMS of PD. The current state of anxiety management (pharmacological methods) is rife with risks, such as falls, excessive daytime sleepiness, and resultant nighttime sleep disruption. Replacement of the current paradigm with the use of self-managed nonpharmacological approaches reduces or eliminates these risks. To this end, most of the interventions studied to date share the use of focused breathing to elicit relaxation. With further research, focused breathing may provide a safe, easily taught, accessible, effective, and translational self-management intervention.

## Figures and Tables

**Table 1:** Self-managed nonpharmacologic anxiety studies.

Authors	Study design	Study aim or purpose	Intervention	Control	Sample size		Anxiety tool	Statistical significance: yes/no
					Treatment	Control		
*Experimental*								
Dobkin et al. [33]	RCT	Secondary	Tailored CBT	Usual care	41	39	HAM-A	Yes
Craig et al. [35]	RCT	Secondary	Listening to relaxing music	NMT	18	14	BAI	Yes
Sajatovic et al. [30]	RCT	Secondary	Self-managed program + exercise	Exercise	12	12	COVI	No
Advocat et al. [38]	RCT	Secondary	Mindfulness-based lifestyle classes	Waitlist	24	33	DASS-21	No
Veazey et al. [40]	RCT	Secondary	Telephone or in-person CBT	Usual Care	4	3	BAI	No
*Quasi-experimental*								
Dobkin et al. [41]	Uncontrolled intervention pilot study	Secondary	Telephone-based CBT	—	20		HAM-A	Yes
Troeung et al. [42]	Nonrandomized intervention	Primary	PD-specific CBT	—	18		DASS-21	Yes
Berardelli et al. [43]	Nonrandomized intervention	Outcome	Group CBT classes	—	7		HAM-A	Yes
Dissanayaka et al. [70]	Uncontrolled mixed-methodfeasibility study	Primary	Tailored mindfulness-basedCT	—	14		GAI	Yes
Dissanayaka et al. [46]	Uncontrolled intervention study	Primary	Tailored CBT (dyad of pts & caregivers)	—	12 = patients		GAI HAM-A IQAD	No/yes/yes Yes (all times) No (all times)
					10 = caregivers			
Dobkin, Allen, & Menza [48]	Uncontrolled intervention pilot	Outcome	Modified & individualized CBT	—	13		STAI	No
Calleo et al. [50]	Nonrandomized intervention study	Secondary	Individualized, telephone, or in-person CBT	—	Patients = 8 telephone 8 in-person		SIGH-A	No
					Caregivers = 4 telephone 2 in-person			
Shinmei et al. [53]	Uncontrolled feasibility study	Secondary	Culturally adapted CBT		18		HADS	No

Note: RCT = randomized controlled trials; N = number; Tx = treatment; NMT = neuromuscular therapy; CBT = cognitive-based therapy; BAI = Beck Anxiety Inventory; HAM-A = Hamilton Anxiety Measure; COVI = Covi Anxiety Scale; DASS-21 = Depression Anxiety Stress Scale-21; GAI = Geriatric Anxiety Inventory; IQAD = Informant Questionnaire for Anxiety in Dementia; STAI = Spielberger State-Trait Anxiety Inventory; SIGH-A = Structured Interview Guide for HAM-A; HADS = Hospital Anxiety and Depression Scale; OASIS = Overall Anxiety Severity and Impairment Scale.
